# The role of the community of purpose in maternal mHealth interventions in Sub-Saharan Africa context

**DOI:** 10.3389/fdgth.2024.1343965

**Published:** 2024-09-12

**Authors:** Karen Sowon, Priscilla Maliwichi, Wallace Chigona, Address Malata

**Affiliations:** ^1^ Department of Information Systems, University of Cape Town, Cape Town, South Africa; ^2^ Department of Computer Science and Information Technology, Malawi University of Science and Technology, Limbe, Malawi; ^3^ Office of the Vice Chancellor, Malawi University of Science and Technology, Limbe, Malawi

**Keywords:** maternal healthcare seeking, digital health, mHealth, community of purpose, social capital, mHealth adoption and use, mobile health, mHealth intervention

## Abstract

**Background:**

mHealth has increasingly been touted as having the potential to help Sub-Saharan Africa achieve their health-related sustainable development goals by reducing maternal mortality rates. Such interventions are implemented as one-way or two-way systems where maternal clients receive pregnancy related information via SMS. While such technologies often view the users (the maternal health client) as having agency to adopt, we know from pregnancy literature that the pregnancy experience in Africa and other developing countries is often more collective. In addition to the maternal health client, other members of the community have high stakes in the pregnancy, and this often affects maternal healthcare-seeking behavior.

**Objective:**

The aim of this paper, therefore, is to understand the pathways through which these other members of the community affect mHealth use.

**Methods:**

The study used a qualitative approach and a case study research design. We analyzed two mHealth cases from Kenya and Malawi. In the Kenyan case, maternal health clients had mobile phones to receive pregnancy-related messages, while in the Malawi case, maternal health clients did not have mobile phones. Data were collected through interviews and focus group discussions. The study used an inductive thematic analysis to analyze the data.

**Results:**

The findings show that maternal stakeholders form a community of purpose (CoP) that plays a crucial role in the implementation, uptake, and use of mHealth. The CoP influences maternal health clients through a diverse range of mechanisms ranging from sensitization, bridging the digital literacy gap and legitimization of the intervention. The nature of influence is largely dependent on the contextual socio-cultural nuances.

**Conclusion:**

Our results provide useful insights to mHealth implementers to know how best to leverage the CoP for better mHealth uptake and usage. For example, engaging healthcare providers could champion adoption and use, while engaging other family-related stakeholders will ensure better usage and compliance, encourage behavior change, and reduce mHealth attrition.

## 1 Introduction

Maternal mortality is still disproportionately high in countries in Sub-Saharan Africa (SSA) compared to more developed nations. The risk of a woman dying is estimated to be 1 in 6 in poorer economies compared to 1 in 30,000 in places such as Northern Europe (1). Developing countries also account for almost all maternal deaths, with almost 70% concentrated in SSA alone (2, 3). The global community has therefore endorsed the reduction of maternal mortality as a critical development goal–with an ambitious target of a mortality rate that is less than 70 per 100,000 by 2030 (4).

Concurrently, the proliferation of mobile phones has led to the rise of mHealth as a potential tool to overcome traditional healthcare barriers and challenges and to meet health-related sustainable development goals. mHealth is characterized as health interventions that employ mobile technologies, including mobile phones, wearable devices, personal digital assistants, tablet PCs, and similar devices. For this study, our definition is limited to the use of mobile phones. The technology leverages the access and portability of mobile phones to provide healthcare services to healthcare consumers (5). The maternal health landscape is filled with projects and studies that demonstrate various applications of mHealth to facilitate point of care, data collection, patient monitoring, and the delivery of health information (6–8). Given the limited technological context of many emerging economies, most mHealth interventions are implemented to disseminate pregnancy-related information, as well as reminders of antenatal care (ANC) visits via short message service (SMS) in one-way or two-way implementations (9). SMS is also the most preferred method to ensure the inclusion of both basic and smartphone users – especially in SSA where approximately 51 percent of mobile phones are smartphones, while 41 percent are basic mobile phones (10). Most of these smartphone owners live in urban areas, while the majority of rural mobile owners own a basic mobile phone (11).

While the use of mHealth shows promise, the first challenge is that interventions are often designed with a single user in mind – a perspective that assumes a one-to-one relationship between an individual and a device such as a mobile phone or a health tracker (12, 13). However, in most contexts in SSA, the ownership of devices does not always follow a one-to-one paradigm. In 2022, the region had a mobile penetration rate of 43 percent (10). This shows that the continent is still lagging in terms of mobile phone ownership, a problem that is often addressed by phone sharing. Furthermore, the gender gap in mobile phone ownership is more pronounced, with women being 13 percent less likely to own a mobile phone than men (10). For example, 44.9 percent of men in Malawi own a mobile phone compared to 37.7 percent of women (14). This gap is especially significant in rural areas, where only 26 percent of women own a mobile phone compared to 47 percent of men (14). In such situations where maternal health clients do not own a mobile phone, access to pregnancy-related information can be through a shared mobile phone in which the owners of these mobile phones can be community health workers (CHWs), family members, friends, community members, or community volunteers (15, 16).

Similarly, even if we were to keep the assumption that all users have mobile phones, studies of health behavior indicate a trend in developing countries to seek advice on treatment from elderly family members (17). In maternal health, the importance of female relatives becomes particularly pronounced, as authority and decision making in pregnancy matters are often socially designated within the female sphere (18, 19). Beyond elderly female family members, the involvement of partners or husbands is significant in the pregnancy journey. In a Muslim society, it was observed that Muslim women were required to seek permission from their husbands for healthcare decisions (20). As decision makers and primary controllers of household resources, men play an essential role in influencing women’s healthcare-seeking behavior (17, 21). Hence, the second challenge lies in the assumption that the maternal health user has complete autonomy to use, or not to use mHealth as would be expected in a more Western context.

These diverse stakeholders that offer social and cultural support to pregnant women form a Community of Purpose (CoP), a term that is inspired by the concept of community of practice that defines a group of people united by a common interest in a specific domain of knowledge (22). The CoP is defined as a community of people working toward a common goal, purpose, or objective (23)—in maternal health, this would be the common objective of a healthy pregnancy (24). The influence that such a group has is often based on the tangible and intangible resources that they share such as shared trust, adherence to group norms and sanctions. In essence, these define social capital (25). In broader terms, social capital entails any instance in which people cooperate for common ends on the basis of shared informal norms and values–which in the context of pregnancy entail all the social and cultural beliefs and norms around pregnancy.

Although behavioral health interventions have often focused on individual-level change, some empirical work has underscored the importance of social capital to health outcomes (26, 27). Given that the importance of other community members to maternal healthcare-seeeking is well established (17, 28, 29), it seems reasonable to conjecture that these influences go beyond traditional healthcare-seeking and also influence the use of mHealth in maternal health contexts. We posited that these relationships are likely to affect mHealth adoption and use. Therefore, research on the dynamics of behavior change in mHealth contexts where social structures such as the CoP are carried out within other sociocultural realities is needed. This understanding is particularly crucial, because, as described before, the pregnancy experience is more collective in Africa than it is in western contexts. Hence, the objective of this research is to examine the impact of the CoP as constructed and determined by gendered norms and sociocultural rules on the success of mHealth use in contexts where users may own phones and where they do not. The questions we seek to answer are:

• RQ1: What role does the CoP play in the adoption and use of maternal mHealth interventions in a Sub-Saharan Africa context?
• RQ2: How does the role of the CoP compare between contexts where users have mobile phones and where they do not?

We used a case study approach to answer these questions with two case studies from Kenya and Malawi. These countries were chosen for two reasons: (1) they both still have high maternal mortality rates and (2) they represent two countries with diverse mobile phone penetration realities which likely influence the use of mHealth interventions. While Kenya’s mobile penetration rate is 117.2 percent, Malawi’s is 57.2 percent (30).

## 2 Methods

The data used in this paper were part of two larger qualitative studies that we conducted separately in Kenya and Malawi. Both studies adopted a case study approach. The Kenyan arm of the study was completed between January and May 2019. The study in Malawi was completed between January and August 2020. This paper reports only on data related to the role of the CoP.

### 2.1 Study design: context and case descriptions

The two cases that we focused on in Kenya and Malawi respectively were the PROMPTS mHealth intervention and Chipatala Cha Pa Foni (CCPF). Given the diverse realities of mobile phones described previously, maternal health clients in Kenya accessed PROMPTS with their own phones. PROMPTS provided staged pregnancy-related information via two-way SMS.

In Malawi, the adoption and use of mHealth in rural settings is hampered by the ownership of mobile phones (11). The price of mobile phones, even the most basic ones, makes it difficult for most people in rural communities to afford a mobile phone due to limited financial resources (31). Other barriers to technology use include poor battery life of mobile phones, network coverage problems, and malfunctioning mobile phone keypad (32). The barrier of non-ownership of mobile phones makes mHealth beneficiaries use borrowed mobile phones to use mHealth interventions. In Malawi therefore, CCPF was targeted at both women with, and without phones. For the purpose of this study, we were interested in how the women without phones in Malawi used mHealth. The following subsections elaborate further on these two case studies:

#### 2.1.1 Case study 1: PROMPTS Maternal mHealth Intervention—Kenya

PROMPTS was initially developed as a postnatal checklist intervention that community health workers were responsible for administering to women after birth, with the aim to encourage the uptake of postpartum care services (33). However, due to the limitations of in-person home visits in a staff-constrained environment, the program evolved into a mobile phone SMS service to reach more women. The postnatal checklist messages were adapted for use as SMS. The messages were also further developed and refined in consultation with the maternal health clients who participated in Focus Group Discussions (FGDs). These sessions aimed to solicit details of the information that women felt they needed after delivery. This was combined with information prioritized by the healthcare provider which they considered pertinent throughout the pregnancy continuum.

The emerging version of PROMPTS was implemented as a toll-free text messaging platform to send staged messages and was combined with a clinician-supported helpdesk to answer questions. Messages were periodically adjusted based on maternal health clients’ feedback and common questions. At the time of the study, the program had since expanded to four other counties (administrative locations in Kenya) had enrolled more than 25,000 women and answered more than 30,000 questions. To create awareness about the intervention, the PROMPTS implementers put up posters in the waiting bays at the respective facilities. Periodically, specific personnel who had been employed by the mHealth implementers visited the facilities to conduct information sessions where they explained to women how the intervention worked and helped those who were interested to register. Registration was done by sending a toll-free SMS with the word ‘MIMBA’ a Kiswahili word for pregnancy, to a specific short code.

#### 2.1.2 Case study 2: the CCPF maternal mHealth intervention - Malawi

The Chipatala Cha Pa Foni (CCPF) intervention which translates to “Health Centre by Phone” in Chichewa was implemented to provide maternal health clients in Malawi with pregnancy tips and reminders. In addition, women could call a toll-free hotline for health information and advice (34). Like PROMPTS in Kenya, CCPF was open to pregnant women, and mothers of infants or children below five years of age who used CCPF while pregnant. At the time of the study, CCPF had enrolled more than 7,500 women and answered more than 2,000 calls per month. Maternal health clients registered for the intervention using their mobile phones, while those without mobile phones registered using mobile phones of family members, community members, or community volunteers. The intervention provided mobile phones to community volunteers, who served as agents of the intervention in their communities. The community volunteers trained the women who did not own mobile phones, on how to access the interventions using the project’s mobile phones. At the time of the study, CCPF was available country-wide and owned by the Malawi Government. More details of the operations of CCPF can be found in (16).

### 2.2 Sampling and recruitment

We used purposeful sampling to recruit study participants. We sampled 40 respondents in Kenya and 31 respondents in Malawi. In Kenya, maternal health clients were recruited by visiting local health facilities, and in addition to participating in interviews, those who agreed to participate in the FGD were also enrolled for the same. In Malawi, two CCPF volunteers were contacted to refer women who accessed CCPF via their phones. Consenting women were contacted mostly through their husbands phones since the women did not own any phones. Similar to Kenya, some women agreed to participate in both interviews and FGDs.

To recruit partners, the researchers reached out to the husbands of consenting women to ask if they were interested in participating in the study. Partners to women in Kenya participated in the FGD only, while in Malawi, partners were interviewed. Although we provide complete sample information for all study participants in Kenya and Malawi Tables A1, A2, we have focused the demographic information on the various individuals who constituted the CoP in the two mHealth contexts Tables A3, A4.

### 2.3 Data collection

Both studies adopted a variety of methods to collect data. However, for this paper, we used data from qualitative interviews and FGDs with various stakeholders in the maternal health context. All interviews lasted 45–60 min, and we audio-recorded them with permission from the participants. The FGDs with 5-8 participants lasted for 60–90 min. Interviews and discussions were conducted in the respective languages: Kiswahili and English in Kenya and Chichewa and English in Malawi. We piloted all data collection instruments with respondents similar to the participant sample and made relevant changes to enhance the clarity of the questions. We do not report on the pilot data in this paper.

#### 2.3.1 Interviews

Researchers from Kenya and Malawi designed the interview questions for their respective countries. In Kenya, the interviews with maternal health clients centered around the following topics:

• their decision-making and considerations to use PROMPTS
• their healthcare-seeking behavior and practices before and after registering for PROMPTS
• what roles other community members played during pregnancy

In Malawi, the interviews with maternal health clients covered the following topics:

• maternal clients’ motivation to use borrowed mobile phones to access the intervention
• cultural issues surrounding pregnancy especially when using a borrowed mobile phone
• the maternal clients’ relationship with mobile phone owners and the other type of support the mobile phone owners offered to maternal clients
• what roles other community members played during pregnancy

The remaining stakeholders in Malawi, that is, community volunteers, members, and health officials, were engaged on the following issues: why they lend maternal clients their mobile phones to use the intervention, what else they did to support maternal clients when using their mobile phones, and how they influenced maternal clients to use the maternal mHealth intervention (Appendix A).

#### 2.3.2 Focus group discussions

The partners of the maternal health clients in Kenya participated in a group discussion where the questions centered around the following themes (Appendix B): their perceptions and attitudes about women using the mHealth intervention, their beliefs and perceptions about pregnancy care and support for pregnant women, as well as their perceptions on the impact of the SMS intervention on women’s lives.

The FGDs with the maternal health clients in Malawi centered on how they used the intervention. The FGDs in both cases were useful in gaining additional insight into culture, social norms, values, and power relationships with respect to maternal healthcare and pregnancy.

#### 2.3.3 Data collection procedures

We sought informed consent before all interviews and FGDs that were conducted in a private space. For the FGDs, the researchers explained the limitations of group confidentiality, but encouraged participants to maintain this confidentiality beyond the meeting. We also assigned pseudonyms to all users to enhance their confidentiality. We were aware of the risk of interviewing pregnant women, that there could be an emergency during interviews and FGDs. Thus, in Malawi, we did not include pregnant women in our sample. However, in Kenya, where most of the maternal health participants were pregnant or early postpartum, the interviews were conducted in a private space at the health facility where the women visited for antenatal care (ANC) or at their homes if they preferred.

### 2.4 Data analysis

The researchers translated and transcribed any non-English data into English. We deidentified all data before analysis as an extra precaution in case participants’ personal information had been captured in the course of the study. The transcripts were then uploaded to Nvivo 12 for analysis. We employed an inductive approach (35) to find patterns in the data (codes) following which we grouped these into higher-level themes and sub-themes (Table 1). Given that the original studies were completed at two different times, the respective researchers individually coded the data with frequent discussions with one other joint researcher to discuss emerging themes and to consider alternative interpretations. These peer discussions helped limit researcher bias. The final themes from this analysis process have been presented in the findings section with selected supporting excerpts of respondents’ verbatim quotes. The deidentified verbatim excerpts are presented using the following pseudonym structure: “*CaseTypeofParticipant numeric number*,” for example, PROMPTSClient1.

**Table 1:** Themes and their descriptions.

Main theme	Sub-theme	Description
Persuasion		The action of encouraging someone to do something
Persuasion factors	Obeying authority	Tendency people have to try to please those in charge
	Maintaining harmony	The act of avoiding fighting or arguing but rather living peacefully
	Peer influence	Doing something because your friend or other people in the community are doing it
Training		Teaching or developing other people’s technical mobile phone usage skills
Technology access		Making a technological device such as a mobile phone available to someone
Sensitization		The process of letting someone know about an event or things that are happening in a community
Technology legitimization		The act of checking if the information is aligned with the Ministry of Health information of someone’s country

The two studies adopted Lincoln and Guba’s (36) model of trustworthiness to ensure rigor. In addition to triangulating the data collection methods, we adopted peer debriefing with a senior researcher to review the inquiry process and cross-check inferences from the data analysis process. We also provide the study context and case description to ensure transferability to other similar contexts.

### 2.5 Ethics approvals

Both studies were conducted in one institution. Therefore, we obtained both institutional and national ethics approval before data collection. In Kenya, we obtained permission from the Amref Research Ethics Committee as well as from the National Council of Science and Technology (NACOSTI). In Malawi, we obtained permission from the Malawi Ministry of Health and Balaka District Health Office. Furthermore, we obtained ethical clearance from the National Health Sciences Research Committee (Malawi). We also sought permission from the intervention implementing agencies in both countries to use these interventions as case studies.

## 3 Results

We interviewed both maternal health clients and various members of the community that make up the maternal health CoP. These include other family members, community members, community volunteers, and health surveillance assistants (HSAs). Tables A3, A4 shows the demographic characteristics of CoP members in the two countries. Our analysis resulted in 5 major themes that highlight how the CoP influences maternal healthcare clients to use maternal mHealth interventions. The five mechanisms that we find are:

1. Persuading maternal clients to use the intervention
2. Training maternal clients on how to use digital health intervention
3. Provision of mobile phone access to maternal clients and
4. Sensitizing maternal clients about the interventions
5. Legitimizing the intervention by corroborating the health information

**Figure 1:**
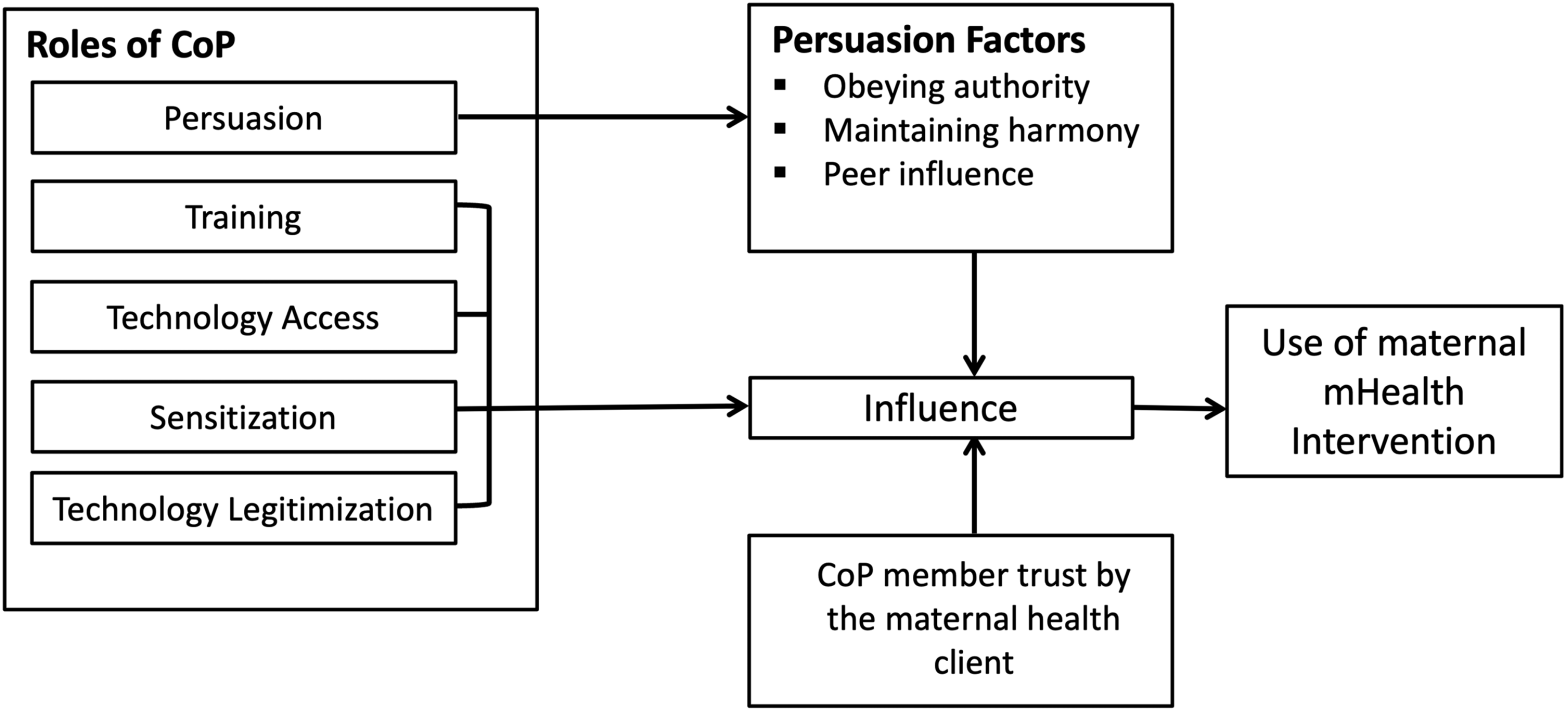
Community of Purpose framework for influencing mHealth intervention use.

We further find that certain factors mediate the role of the CoP as illustrated in Figure 1. Persuasion was further mediated by factors that, in combination with trust between maternal clients and members of the CoP, played a role in influencing the use of mHealth.

### 3.1 Persuading maternal clients to use mHealth

Maternal clients in this study were convinced to register for mHealth interventions by other members of the community, such as HSAs, community volunteers, and their husbands. Although maternal clients in Malawi were persuaded to use the CCPF mHealth intervention by HSAs and community volunteers who visited them at their homes or met at social gatherings in their communities, the persuasion of women in Kenya happened through the implementation representatives of the intervention who visited the health facilities to provide information sessions.

“We had to convince [maternal health clients] that the intervention was legitimate and that it is something approved by the county… their trust issue, they said, was that different people, or conmen or scammers send them messages [referring to general mobile phone scams] of which they don't know if they are true or not.” [PROMPTSInformant 3]

Persuasion is a fundamental element in changing human behaviour and attitude. During persuasion, maternal clients were informed about the mHealth intervention and its benefits, which in turn led to a willingness to adopt.

“I joined [CCPF] because of the advice I received from the HSA, that I can be helped while at home. Also, I can be listening to messages about my pregnancy…and when I deliver; I can also follow how my baby is growing” CCPFClient 13.

PROMPTSClient 20 said: “What motivated me to register was that I might be in the house, and I encounter a particular challenge, and maybe I don't have money at that moment… to reach the hospital. [So], while I am sitting in my house, I can send a message telling them I'm experiencing this and the other.”

The findings also indicate that before the CoP could encourage mothers to use mHealth interventions, they themselves had to be convinced of its perceived usefulness. For example, in the case of the mHealth intervention in Kenya, one partner referring to the wife said: “I was very happy because she told me that she knew about [the intervention] at the hospital and that made me comfortable because I knew that the hospital could not put up posters if the information was not genuine” PROMPTSMenFGD.

Previous research (9, 37) has shown that when such health interventions are associated with a trusted entity, they engender a higher level of trust for both the CoP and the maternal health client and subsequently encourages adoption. One participant said “My husband asked me about these messages. I told him it’s something like an online clinic through our phone, and that the doctors were from XYZ Hospital. And then he said it was okay.” PROMPTSClient 10. In both Kenya and Malawi, the health facilities, healthcare providers and community volunteers represented trusted entities as seen in what one participant shared: “I remember that my husband was concerned [about me meeting you]. ‘Where will you be meeting?’ I told him that it would be at the hospital. And he said that was okay” [PROMPTSClient 15].

Trusting the intervention subsequently influenced the the actions of other stakeholders. A partner to one of the women explained how the mHealth intervention changed their perceptions and behavior. “The other thing is about nutrition, because maybe we had that old mentality about what pregnant women should eat, but now after interacting with this SMS service, you get to know other nutritional meals that pregnant women can eat” PROMPTSPartner2.

We identified three persuasion factors through which persuasion operates: (i) obeying authority, (ii) maintaining harmony, and (iii) peer influence.

#### 3.1.1 Obeying authority

Our findings highlight that maternal health clients in both Kenya and Malawi easily deferred to figures in positions of authority. For example, since HSAs and healthcare providers were generally considered highly respected, maternal clients accepted and appropriated CCPF and PROMPTS when recommended by these higher figures. One key informant explained this better. “If a provider tells [the mother], you’re good, go home, it’s unfortunate that still very few people can oppose a provider or give their own opinion even if they feel it’s not right…” [PROMPTSInformant 1]. The power wielded by healthcare providers was also captured by PROMPTSClient21 who said: “[My husband] used to tell me to go to the older woman and then I asked in the SMS and they told me that wasn’t useful, the doctor has the ability⋯ [my husband] just agreed and said that the doctor is educated than the older women.”

The male partners in the study corroborated the idea of submitting to authority. In addition to healthcare providers, other stakeholders, such as older women, also exercised authority. These women generally provided care and oversight of a woman’s pregnancy, their advice was usually upheld. “We get [information] from those [older mothers] who have delivered and know more than you. Maybe they have other children and they have experienced this before and so they know what you don’t know” PROMPTSClient13. The men in FGD in the Kenyan case said that when it came to pregnancy-related advice that was given by these older women, they “Did not [question] the reasons, but followed the [advice] because there could be a reason why and you don’t want to go against what you have been warned” [PROMPTSMenFGD].

#### 3.1.2 Maintaining harmony

Some maternal health clients were forced to use interventions because they wanted to maintain harmony with the respective members of the CoP. “My husband did the registration, so I didn’t know what they were talking about. However, when the messages came, we were reading them together…” [CCPFClient 1]. Such compliance to maintain harmony may have been necessitated by the fact that most of the maternal clients depended on their partners for financial support. The women in the FGD group in Kenya said: “They [husbands] are the heads of the family, and we need financial assistance, so you inform them so that they can give you money.”

“We’re finding that the [husbands] are the ones who are ensuring that the woman is eating the right diet during pregnancy because most of the times husbands are the providers, especially in our setting… where most women stay at home, and the husbands go to work. So, they’re basically the providers” [PROMPTSInformant 1].

#### 3.1.3 Peer influence

Maternal clients were convinced to use CCPF and PROMPTS because other women were using it. One participant shared that she saw other maternal healthcare clients using CCPF and heard about other maternal clients’ experiences with CCPF which was good. This is similar to findings of other studies who found that mentors and role models in communities have the potential to persuade other users to accept and use the mHealth intervention (38).

### 3.2 Training maternal clients on how to use the mHealth intervention

During the feasibility study before CCPF was implemented, the project implementers found that the women in Malawi had lower literacy than their male counterparts (39). Hence, community members were trained as community volunteers so that they could provide training to maternal clients on how to use the intervention. Training maternal clients on how to use the mHealth intervention promoted social learning and social acceptability of the intervention among members of the community. With the lack of digital skills likely to disproportionately affect users without technologies such as mobile phones, such community-enabled digital skills training was central to mHealth adoption and use. Thus, training these maternal clients on how to use CCPF on a mobile phone was the first step to influence the use of the mHealth intervention. Otherwise, the non-usage of mHealth interventions could lead to their failure to achieve their purpose (40), which is to improve the maternal healthcare-seeking bahavior.

### 3.3 Technology access

Maternal clients who stand to benefit from mHealth could be disproportionately disadvantaged by the lack of mobile phones. Access to a mobile phone is an important prerequisite for the success of mHealth use. Although maternal clients in Malawi did not own mobile phones, they accessed a mobile phone through family circles such as their husbands and mothers-in-law, as well as community volunteers and community members. “For CCPF, I use the community member’s mobile phone. I dial the CCPF number myself and I can talk to the hotline worker. I can talk privately on the mobile phone. I am used to this mobile phone for CCPF because this mobile phone is always available to me…” [CCPFClient 15].

A community volunteer mentioned that her mobile phone was available for maternal clients to use for CCPF and she even visited registered and unregistered maternal clients to see if maternal clients needed to use her mobile phone to access CCPF. Another volunteer mentioned that after the mobile phone that she was given during the pilot phase of the intervention malfunctioned, she bought another phone and continued her role as a community volunteer in her village. “The mothers live far from where I live, so I just choose a weekday that I can visit the mothers…” [CCPFCommunity Volunteer 1].

One CCPF community member, a wife of a village headman, said she allowed maternal clients to use her mobile phone to access the intervention. In the rural context of Malawi, a village headman presided over various aspects of life, especially cultural and social issues in the community (41). Village headmen were also expected to advise and support their community, as well as to implement policies handed down from the national level (42).

### 3.4 Sensitizing maternal clients

In the case of CCPF, community volunteers and HSAs were mandated to sensitize maternal healthcare clients about the maternal mHealth intervention in their communities. Community volunteers paid special attention to maternal clients who did not own a mobile phone to alert them about CCPF. “The health counselor in the village told us that there is CCPF, and you can call them anytime to ask about any pregnancy-related problems…” [CCPFClient 8]. Furthermore, husbands of maternal clients attested that when they received an SMS about CCPF, they informed their wives about it. In this study, male participation in maternal-related issues was more prevalent, especially in sensitizing maternal clients about CCPF and persuading maternal clients to use CCPF.

In the case of PROMPTS, the sensitization was performed by PROMPTS personnel who visited the specified clinics on certain days of the week to explain the intervention to the waiting ANC clients while encouraging them to enroll. In this case, these personnel also helped reduce any uncertainties by explaining to the women what the intervention was about and explaining that it was free to use.

### 3.5 Technology legitimization

The CoP facilitated the legitimization of the mHealth intervention by offering a means for maternal health clients to corroborate the information they received from both PROMPTS and CCPF. Given the diverse uncertainties around pregnancy emanating from the cultural context, maternal healthcare clients established the credibility of pregnancy-related information to establish trust. They achieved this by comparing the mHealth-related information with the advice of trusted stakeholders, including trusted older female relatives and sometimes healthcare providers, when they attended ANC clinics. The legitimization itself was achieved by: (i) Comparing new information with existing knowledge and (ii) Comparing information across multiple sources.

Legitimacy is defined as “a generalized perception or assumption that the actions of an entity are desirable, proper, or appropriate within some socially constructed system of norms, values, beliefs and definitions” (43). The women determined the reliability of the information against a priori knowledge. “At times there are some questions you sort of already have the answers to, but you just ask to make a comparison” [PROMPTSClient 27]. When one trustworthy source was not available, the women established the credibility of the information by comparing across multiple sources: “I had two sources of information, the SMS and the doctors at the clinic. After receiving the messages I would verify some of that information by asking the doctor the same questions, which led to total trust because I saw that the information was accurate” [PROMPTSClient 10].

Other researchers (44, 45) have noted similar behavior of health clients engaging multiple sources of information and care-seeking to address health concerns.

### 3.6 How does the role of the CoP compare between settings where users have mobile phones compared to where they do not?

Our findings show that the CoP plays an important role both when users have a mobile phone and when they do not. However, the exact nature of their role is sometimes dependent on the unique circumstances of the maternal clients. For example, in Malawi where mHealth users did not have phones, in addition to providing technology access, the CoP sensitized women on the existence of the intervention and acted as important influencers to adoption and use. Thus, the CoP played a more direct role. In Kenya, where women already had phones, the CoP had a more indirect influence. For example, by providing opportunities for women to corroborate the information they received from the mHealth platform, CoP acted as indirect facilitators of adoption. We therefore observe that although the specific mechanisms of the CoP’s influence varies between contexts, their centrality within the mHealth appropriation and use domain remains the same.

## 4 Discussion

The research community has called on the need for studies to provide a detailed understanding of how social capital can be manipulated to influence health results (46). We evaluate the role of the community of purpose as a vehicle through which social capital can manifest. The findings of this study suggest that CoP played different roles in influencing maternal clients to use maternal mHealth interventions. Collectively, we draw three important lessons from our findings: (1) The extent of CoP influence is inextricably linked to the cultural context, (2) The involvement of the CoP catalyzes health behavior change by providing multiple layers of influence, and (3) While the CoP as a whole is central to maternal health, the respective constituents do not wield equal power.

### 4.1 Lesson 1: the degree of influence of CoP is inextricably linked to the cultural context

In collectivist cultures, the community is valued over the individual. “Umuntu ngumuntu ngabantu” which loosely translates as “[a] person is a person bacause of other people” (47) gives priority to empathy, caring and understanding, and the contributions of individuals in a community are valued and cherished (31). Furthermore, sharing and neighborly assistance are part of an African identity (48), and are attributed to the Ubuntu philosophy practiced in the communities we studied. Although project implementers in Malawi encouraged maternal clients to use their family member’s mobile phones, as well as friends’ mobile phones, this practice was acceptable due to the Ubuntu value system. These contributed to establishing an inclusive community that helped promote the participation of all members of the community and create a sense of belonging (49, 50). Inclusive communities acknowledge that all members of the community, including the marginalized, should participate in everything that happens in their community (51). The most important thing in an inclusive community is to recognize that people have different needs and that diversity should be valued (51). Therefore, inclusive communities played a vital role in identifying and removing barriers to community participation. When communities are inclusive, sharing of resources, such as mobile phones, helps to reduce the digital divide gap (52), thus making mHealth interventions work in resource-constrained communities.

In addition to the Ubuntu value system that facilitated certain sharing-based technology outcomes, the persuasion factors are rooted in culture. Obedience to authority, seeking harmony, and peer influence are dependent on aspects of culture such as power distance and norms in collectivist cultures. Hence, we do not expect that the same influence factors will work in the same way in, say, more individualistic cultures.

### 4.2 Lesson 2: the CoP acts as agents of change in the context of maternal mHealth interventions

By influencing the use of mHealth among the women, offering training, increasing the accessibility and reach of mHealth and facilitating the legitimization of mHealth for continued use, the CoP acts as agents of change. It has been previously noted that the low literacy of maternal healthcare clients inhibits maternal mHealth intervention designed for poor-resource settings (53, 54). ICT capabilities are essential for the uptake of many digital technologies including mHealth interventions. In this study, to promote ICT usage skills, community volunteers trained maternal healthcare clients on how to access CCPF using a mobile phone. By providing the technology by which women could have access to maternal health information, the CoP played an important role of being infomediaries.

Maternal mHealth interventions also translate to desired health outcomes only as health-related advice is followed. In Malawi, CCPF community volunteers visited maternal clients in their homes to inform and encourage its use. In Kenya, once legitimized and women’s use of the intervention became a culturally appropriate behavior, other members of the CoP were able to facilitate follow-through with information that would previously be impossible, such as those related to food taboos that husbands would not purchase for their wives. Other studies have similarly shown that various actors play an important role as agents of behavior change (55, 56). Community members are particularly effective because they live within the proximity of maternal clients. This means that they can more easily monitor the behaviors of their maternal healthcare clients to ensure that they are following the information appropriately. Other studies have called these CoP members ’watchdog-oriented community members’ because they play an important supervisory function in society by monitoring the health of consumers in their communities (57).

Similarly, initial trust is necessary for the adoption of maternal mHealth services, especially given that the maternal healthcare-seeking context contributes to the increased liability for the newness of new interventions which negatively impacts adoption (24, 37). Various design and implementation characteristics that help minimize perceived risks and uncertainties about using a new mHealth intervention can help to engender initial trust (37). By providing an avenue through which women legitimize the intervention and therefore overcome uncertainties related to the newness of the intervention, the CoP acted as agents of change to increase adoption.

### 4.3 Lesson 3: although CoP as a whole is central to maternal health, the respective constituents do not wield equal power

The findings confirm that the CoP is central to the experience of using maternal mHealth interventions. However, we find that CoP members play different roles, and we posit that some of these differences arise from their respective social capital as individual constituents. For example, health care workers wield more power as “gate-keepers” of medical care based on their expertise, training, and education. However, older women provide emotional and domestic support during pregnancy, and the power they exercise is based on age and perceived experience with pregnancy-related issues. Finally, other stakeholders like partners are often considered the main heads of their households, decision makers, and financial controllers. Some of these roles have been highlighted before in literature on maternal health, but our study illustrates that these different aspects come together to form a complex web of dependencies that influence mHealth usage. In a sense, we show that these aspects of power and social structures also translate to the adoption and use of digital health technology.

### 4.4 Study limitations

Although we had planned to engage a diverse group of other members of the community closely involved in woman’s pregnancy in Kenya such as their female relatives: mothers, grandmothers and mothers-in-law, this was not possible because most women in the urban areas lived away from their next of kin most of whom were in their rural places of origin. We believe that a broader range of insights from these key stakeholders would be valuable in enriching the findings of this study. The context of pregnancy may also be nuanced when compared to other health domains, and therefore we claim no generalizability of the findings to other areas where change in health behavior may be a desired outcome. Other limitations relate to the inherent nature of qualitative research. First, given the small sample size, our results may not be generalizable to entire populations of maternal mHealth users. Second, some nuances may also have been lost in translation of the interviews between the languages, which we mitigated by having the researchers from Malawi and Kenya do the transcription to English. Third, the self-reported nature of the interviews may add additional biases. However, we believe that the participation of multiple participants facilitates a better triangulation and validity of the findings.

## 5 Recommendations and future directions

Our study offers an in-depth understanding of the centrality of other community members in the context of a woman’s pregnancy (which we have referred to as Community of Purpose -CoP) in the adoption and use of digital health technologies among maternal health clients. The cases we have drawn from are maternal health interventions that were implemented in Kenya and Malawi among low socioeconomic women and among women without phones, respectively. Our study is qualitative and we do not make any claims on generalizability of the findings. However, we hypothesize that the insights generated may be relevant to other African countries that share similar contexts both economically and culturally.

We offer the following two recommendations.

### 5.1 Recommendation 1: design and implementation of mHealth and other similar digital health technologies with the different roles of the CoP in mind

The study underscores the need for implementers and developers of such technology to consider more holistically the various relationships and the different roles they play and design and implement technology in a manner that will maximize on outcomes by leveraging their respective roles and minimize unnecessary adoption and usage challenges. For example, in the CCPF, the recruitment and training of community volunteers proved to be invaluable. Although this may have been necessary due to the unique context of mHealth users without phones, we posit that this would be equally useful in contexts where users have phones. The findings of both cases demonstrate that associating mHealth interventions with an entity that the community already trusts (for example, hospitals and healthcare workers) contributes to the initial legitimization of mHealth interventions, which facilitates the adoption of mHealth. Since the CoP is also central to legitimizing information by offering a means for maternal health client to corroborate the information they receive, involving them in the design and implementation of digital health interventions will prove extremely useful in contexts such as those presented in this study. Although the involvement of healthcare providers in the implementation of mHealth can be beneficial in encouraging adoption, it has potential drawbacks. Patient dissatisfaction with providers involved in health interventions could negatively affect their use. Possible solutions involve managing the intervention’s operations independently, but involving healthcare workers to create awareness. This will also ensure that providers in resource-constrained settings do not feel burdened by the additional processes of an intervention, and allow patients who prefer the anonymity of mHealth interventions to leverage on such affordances. Involving other CoP members, such as volunteers for women without phones, would also have to think about how to increase motivation. This might entail some form of compensation for their time as seen in Larsen-Cooper et al., (58). Given that our study was limited to the domain of maternal health, we encourage other researchers to explore the role of the CoP in other health domains and for various health outcomes.

### 5.2 Recommendation 2: conduct further research to establish when group targeting of interventions is beneficial to individual targeting

Given that many maternal healthcare-seeking behaviors occur within the purview of social and cultural norms within the society, interventions at the relevant community level might yield more significant outcomes than individual-focused interventions. Our research supports the idea of group (CoP) targeted interventions in maternal health in Africa. More empirical research would be useful in establishing this. Future research could contribute to building a tool that can be used for context assessment and as a guide to know when and where group or individual targeting would be more desirable.

## Data Availability

The raw data supporting the conclusions of this article will be made available by the authors, without undue reservation.

## Appendix A. FGD interview questions for the CoP in Kenya

**The questions below will be used to engage the SOs to gain further insights on power dynamics and decision making process in the mHealth environment that affect utilization of mHealth interventions**

Describe your understanding of the pregnancy experience:

• What is the definition of a good/successful pregnancy experience?
• What rules and guidelines exist in your community/family/society to make this goal possible?

Please share about the traditional methods/your previous ideas of pregnancy-related support

• Before learning from the SMSs or from the health facility, what were your ideas on:
  – the role of the healthcare provider during pregnancy
  – when a pregnant woman should start seeing the healthcare provider
  – how many visits to the doctor during pregnancy?
  – the procedure for having her pregnancy related questions answered: who can she talk to and what informs who she can talk to?
• When in the pregnancy are these people involved? (pregnancy stage or circumstances necessitating involvement)
  – Among the people who can be involved, who is responsible for what?
• Who is responsible for the decision of when, where and how a pregnant woman seeks care and support? (if not husband, what is the role of the husband in this decision making process?)
• What is the decision making role/authority based on?

How else can a mother within your society achieve a successful pregnancy?

• What is the role of other alternative sources of care like TBAs in the pregnancy care system?
• What do think about your partner following all that the intervention prescribes? Are there things you felt it necessary to seek a second opinion to confirm what you received from the SMSs?
• Please describe what you see as your role in the decisions regarding following/adhering to what the intervention though the SMSs would prescribe.

What challenges have you experienced with the used of the SMS service for pregnancy support? (Any conflicts in the nature of information, the asking/answering of questions etc.)

• How agreeable are you to her following everything the intervention says?
• Please describe your process of resolving conflict if what the SMS says is different from what you knew before.

How do you feel the SMS program has changed the healthcare seeking experience for you and your partner?

• Did the use of the SMSs by your partners change your place and role in the healthcare seeking decision making process? How? How did you feel about this?
• How did the SMS program change your involvement and engagement with the pregnancy?
• How did the SMSs change your interaction with the healthcare providers?

**Table A1:** Study sample information for PROMPTS intervention.

	Women	Significant other	mHealth official	Healthcare providers
Population and sample size (n)	30	5	3	2
Study procedures	Interviews and FGDs	FGD	Interviews	Informal discussion

How else would you say the SMSs have transformed the pregnancy experience for you as a partner?

**Table A2:** Study sample information for CCPF intervention.

	Women	Significant other	mHealth official	Hotline worker and community volunteer
Population and sample size (n)	20	6	2	3
Study procedures	Interviews and FGDs	Interviews	Interviews	Interviews

**Table A3:** CCPF CoP demographics.

Mobile phone owner	Age	Gender	No of maternal clients who used the phone
Husband 1	39	M	1
Husband 2	20	M	1
Husband 3	37	M	1
Husband 4	37	M	1
Mother-in-law 1	35	F	1
Community volunteer 1	42	F	8
Community member 1	43	F	7
Officer 1–3	–	M	–

**Table A4:** PROMPTS CoP demographics.

Respondent	Partner to	Age	Education	Work status
Partner 1	Mother 20	27	Primary	Casual laborer
Partner 2	Mother 24	29	Primary	Casual laborer
Partner 3	Mother 17	35	Tertiary	Teacher
Partner 4	Mother 12	30	Secondary	Self employed
Partner 5	Mother 9	28	Secondary	Casual laborer
mHealth officials 1–3	–	–	–	–
Healthcare providers 1–2	–	–	–	–

## Appendix B. Semi-structured interview for the CoP (mobile phone owners) in Malawi

The questions below will be used as a guide for the interview with mobile phone owners (CHWs, family members, friend). The question may be rephrased and probed in various ways.

**Relationship with the maternal healthcare client**

1. What is the relationship with the maternal healthcare client?
  a. Client
  b. Mother
  c. Wife
  d. Friend
  e. Other
2. Besides the intervention, what other purposes do you allow people to use your phone?
3. How do you decide which people to use your phone?
4. How long is the maternal healthcare client being using your phone?
5. What other roles did you do to make sure that mothers use the intervention?

**Motivation to let the maternal healthcare client their mobile phone**

6. Why do you allow maternal healthcare client to use your phone?
7. Describe the process how maternal healthcare client asks to use your phone?
8. Describe the process how someone asks to use the phone for normal use?

**Benefits and challenges**

9. Why do you think other people do not allow maternal healthcare clients to use their phones?
10. What do you get in return when maternal healthcare clients are using your phone?
11. What challenges do you face when maternal healthcare clients are using your phone?
